# Association Between HbA1c Levels and Postpartum Depressive Symptoms: Insights from Edinburgh Postnatal Depression Scale Screening After Gestational Diabetes Mellitus

**DOI:** 10.3390/jcm15155919

**Published:** 2026-07-29

**Authors:** Sophia Lutz, Yvonne Lindemann, Ekkehard Schleußner, Tanja Groten, Friederike Weschenfelder

**Affiliations:** 1 Department of Obstetrics, University Hospital Jena, Friedrich Schiller University, Am Klinikum 1, 07747 Jena, Germany; sophia.lutz@uni-jena.de (S.L.); yvonne.lindemann@med.uni-jena.de (Y.L.); ekkehard.schleussner@med.uni-jena.de (E.S.); 2 Department of Obstetrics, Faculty of Medicine, University Hospital Cologne, University of Cologne, Kerpener Straße 34, 50931 Köln, Germany; tanja.groten@uk-koeln.de

**Keywords:** gestational diabetes mellitus, postpartum depression, HbA1c, EPDS

## Abstract

**Background/Objectives**: Depressive symptoms are more frequent following pregnancies complicated by gestational diabetes mellitus (GDM), prompting postpartum depression screening recommendations in the German S3 guideline. Although GDM is associated with increased postpartum depression risk, the relationships between postpartum metabolic parameters and the Edinburgh Postnatal Depression Scale (EPDS) scores remain unclear. We investigated whether metabolic markers measured during diabetes screening 6–12 weeks postpartum are associated with depressive symptoms. **Methods**: We retrospectively analyzed EPDS screenings conducted during postpartum testing at our inpatient clinic between 07/2021 and 02/2024. The EPDS is a validated 10-item tool (score range 0–30), with scores ≥10 indicating potential depression. Metabolic parameters included HbA1c, the 75 g oral glucose tolerance test (oGTT), insulin, and the Homeostasis Model Assessment index (HOMA index). Group comparisons were performed using non-parametric tests and receiver operating characteristic (ROC) analyses, with the Youden index identifying optimal cutoffs. **Results**: Among the 102 women presenting for postpartum testing, 43.1% had normal glucose tolerance, 54.9% prediabetes, and 2.0% diabetes. A total of 13.7% (*n* = 14) screened positive for potential depression, including two who reported suicidal ideations. Women with positive EPDS scores had significantly higher HbA1c [5.7% (38.3 mmol/mol) vs. 5.4% (35.5 mmol/mol), *p* = 0.013]. An HbA1c cutoff of 5.42% (35.75 mmol/mol) showed discriminatory ability for depressive symptoms with an Area Under the Curve (AUC) of 0.708 (CI 0.567–0.849). Other metabolic parameters showed no significant associations. **Conclusions**: Elevated postpartum HbA1c is associated with depressive symptoms after GDM pregnancies. An HbA1c threshold of 5.42% (35.75 mmol/mol) may help identify women at increased postpartum depression risk and prompt subsequent psychological assessment when depression screening is otherwise omitted.

## 1. Introduction

Gestational diabetes mellitus (GDM) is one of the most common metabolic complications during pregnancy, affecting up to 16% of individuals worldwide, depending on the diagnostic criteria applied, ethnicity, and geographical region [1]. Postpartum depression, defined as the presence of depressive symptoms within the first weeks to months following childbirth [2,3], has a global prevalence of approximately 17% and can have severe consequences for the mother’s mental and physical health, which in turn may negatively affect the child’s cognitive, emotional, and behavioral development [3,4].

Both conditions have gained increasing attention in clinical and epidemiological research, with numerous studies suggesting a potential association between GDM and postpartum depression [5], as well as broader links between diabetes mellitus and depression outside the context of pregnancy. Proposed mediating mechanisms underlying this complex and reciprocal relationship include hormonal changes, inflammation, and psychosocial stressors [1,6,7]. Given the increased prevalence of depression following pregnancies complicated by GDM, the German S3 guideline on GDM recommends depression screening using the Edinburgh Postnatal Depression Scale (EPDS) 6–12 weeks following childbirth [8,9].

Nonetheless, screening for postpartum depression in GDM patients has not yet been incorporated into most international postpartum diabetes screening guidelines. In Germany, only approximately 40% of women attend postpartum follow-up screening [10]; and among these, systematic screening for depression is not consistently implemented.

However, previous research on the association between GDM and postpartum depressive outcomes shows substantial methodological heterogeneity [5], often relying on clinical diagnoses or self-reported medical histories of depressive symptoms or even depression without a detailed analysis of physiological parameters. Moreover, most available studies have focused on the epidemiological association between GDM and postpartum depression, whereas the potential role of routinely assessed postpartum metabolic parameters remains insufficiently explored, and studies capturing this relationship of depressive symptoms and metabolic parameters at a clearly defined point in time during or after pregnancies with GDM remain scarce.

The aim of this study was to investigate whether an association can be identified between metabolic parameters assessed during routine postpartum diabetes screening following pregnancies complicated by GDM and EPDS scores indicating the severity of depressive symptoms. Specifically, we examined HbA1c, glucose levels, insulin, and the Homeostasis Model Assessment of Insulin Resistance index (HOMA index), while accounting for potential confounding factors to enable a more nuanced understanding of the complex interactions between metabolic changes and mental health in the postpartum period.

Among the routinely assessed metabolic markers, HbA1c is of particular pragmatic interest in this context and is distinct from glucose and insulin measurements, as it is routinely assessed during postpartum metabolic follow-up and reflects glycemic exposure over the preceding weeks rather than a single measurement.

Especially in routine clinical settings where systematic postpartum depression screening is still not consistently implemented, we explored whether HbA1c may serve as a pragmatic complementary marker to identify women in whom a missed guideline-recommended EPDS screening should be performed without delay.

We hypothesized that a direct association could be identified between metabolic parameters such as glucose levels, insulin, HbA1c, and the HOMA index and EPDS scores indicating the severity of depressive symptoms following pregnancies complicated by GDM.

## 2. Materials and Methods

### 2.1. Study Population

**Figure 1:**
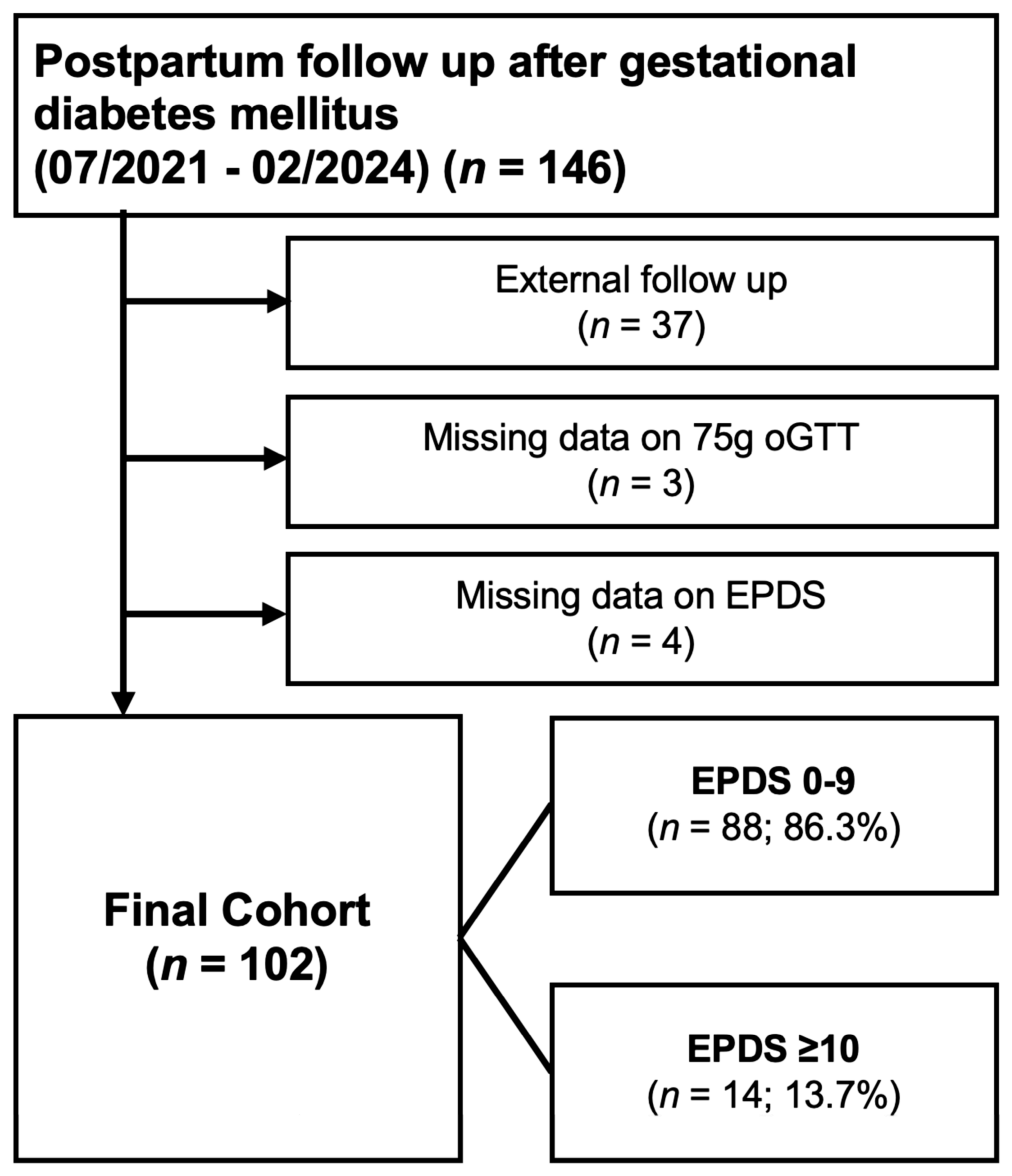
Flow diagram of participant inclusion and study cohort. Of 146 women who attended postpartum follow-up after gestational diabetes mellitus (GDM) between July 2021 and February 2024, 102 met the inclusion criteria and were included in the final analysis. Participants were stratified according to the Edinburgh Postnatal Depression Scale (EPDS) score into EPDS 0–9 (*n* = 88, 86.3%) and EPDS ≥ 10 (*n* = 14, 13.7%).

Between 2021 and 2024, a total of 464 patients with GDM were cared for at our inpatient clinic for GDM. Diagnosis was based on the criteria of the International Association of Diabetes and Pregnancy Study Groups (IADPSG) and WHO-13 criteria [11,12,13,14,15]. The study cohort comprised patients receiving guideline-based standard care at our institution. No additional sampling strategy was applied. Patients were enrolled as part of routine clinical care, and the initial study cohort was restricted to those who attended the scheduled follow-up examination, which is part of the standard-of-care management according to current clinical guidelines [8,9]. All participants fulfilled the predefined eligibility criteria and provided informed consent before inclusion in this study. Patients were primarily not included if postpartum follow-up after GDM was missing (*n* = 318). Of the 146 women attending postpartum follow-up, some were excluded if the screening was conducted exclusively at an external facility (*n* = 37), if available follow-up data for the 75 g oral glucose tolerance test (oGTT) (*n* = 3) or EPDS (*n* = 4) were missing (see Figure 1).

In total, 102 patients with a history of GDM received depression screening using the EPDS 6–12 weeks after delivery, in accordance with the recommendations of the German S3 guideline on GDM [8]. All 102 patients underwent a 75 g oGTT at the time of postpartum follow-up, conducted under standardized conditions recommended by WHO guidelines using venous plasma samples [16]. In 101 patients, additional blood samples were collected to assess multiple metabolic parameters. The 101 participants represent a subset of the total cohort (*n* = 102), as individual metabolic parameters were not available for all patients. Ethical approval for this study was obtained from the Ethics Committee of Friedrich Schiller University in Jena, Germany (Reg. No. 5280-09_17).

### 2.2. Study Data Collection

Patient characteristics were obtained from medical records. A complete-case analysis was performed according to predefined exclusion criteria. Participants with missing data for variables required for the respective analyses were excluded, and no imputation of missing values was performed. Study data were collected and managed using REDCap electronic data capture tools hosted at the Department of Obstetrics, University Hospital Jena [17,18,19]. REDCap (https://projectredcap.org/) (Research Electronic Data Capture) is a secure, web-based software platform supporting data capture for research studies, providing an intuitive interface for validated data capture, audit trails for tracking data manipulation and export, automated export procedures for data downloads to common statistical packages, and procedures for data integration and interoperability with external sources.

Maternal characteristics assessed included age, body mass index (BMI) at follow-up, marital status, educational level, employment status, and smoking. Regarding metabolic control during pregnancy, a distinction was made between patients managed with insulin and those managed with dietary intervention only. Additional information was collected on mode of delivery, neonatal outcomes, and breastfeeding status at follow-up. Metabolic parameters were assessed as part of the routine guideline-based postpartum follow-up visit conducted 6–12 weeks after delivery. As part of the follow-up visit, 75 g oGTTs were performed. During the same appointment, relevant metabolic parameters were assessed, including HbA1c, insulin, C-peptide, and hsCRP. Furthermore, the EPDS questionnaire was administered during the same visit. Data collection, including administration of the EPDS, was performed by a certified diabetes advisor under physician supervision. A predefined protocol required immediate physician consultation whenever clinically relevant abnormalities or concerns, especially including positive responses to item 10 of the EPDS indicating possible suicidal ideation, were identified to discuss the screening results and assess the need for further intervention. When clinically indicated, patients were directly referred to the clinic psychologist for comprehensive psychological evaluation and appropriate follow-up care. Participants completed the questionnaire in a private and quiet clinical setting during the routine postpartum follow-up visit in the outpatient clinic of our obstetric department to ensure confidentiality and minimize distractions.

HbA1c was determined using the Roche Tina-quant Hemoglobin A1cDx Gen. 3 assay (Roche Diagnostics International AG, Rotkreuz, Switzerland) on the cobas c 513 analyzer (Roche Diagnostics International AG, Rotkreuz, Switzerland). The assay is standardized according to the International Federation of Clinical Chemistry (IFCC) reference measurement procedure and is certified by the National Glycohemoglobin Standardization Program (NGSP). According to the manufacturer, the reference interval for healthy adults is 4.0–6.0% (20–42 mmol/mol) [20]. The analytical method was based on a turbidimetric immunological inhibition assay (TINIA) for hemolyzed whole blood [20] (Roche Diagnostics). HbA1c values were DCCT-adjusted (Diabetes Control and Complications Trial). The reference values provided in the system information of the laboratory test applied exclusively to adults, with no pregnancy-specific ranges indicated.

Glucose metabolism was classified according to the 2022 Clinical Practice Recommendations of the German Diabetes Association (DDG) [16]. Normal glucose tolerance (NGT) was defined as a fasting plasma glucose (FPG) < 5.6 mmol/L (<100 mg/dL), a 2-h plasma glucose during a 75 g oGTT < 7.8 mmol/L (<140 mg/dL), and HbA1c < 5.7% (<39 mmol/mol). Prediabetes was defined as impaired fasting glucose (FPG 5.6–6.9 mmol/L [100–125 mg/dL]), impaired glucose tolerance (2-h oGTT plasma glucose 7.8–11.0 mmol/L [140–199 mg/dL]), and/or HbA1c 5.7–6.4% (39–47 mmol/mol). Diabetes mellitus was defined as FPG ≥ 7.0 mmol/L (≥126 mg/dL), 2-h oGTT plasma glucose ≥ 11.1 mmol/L (≥200 mg/dL), random plasma glucose ≥11.1 mmol/L (≥200 mg/dL), or HbA1c ≥ 6.5% (≥48 mmol/mol), in accordance with the DDG recommendations.

Hemoglobin levels were measured at postpartum follow-up to evaluate persisting peripartum anemia.

The HOMA index [21,22,23] was calculated as a quantitative measure of insulin resistance using fasting insulin and glucose values obtained at the time of postpartum follow-up using the following equation:

$$HOMA-IR=\;\frac{insulin\left(mU/L\right)\times glucose(mmol/L)}{22.5\;}$$

### 2.3. Study Questionnaire—EPDS

The risk of postpartum depression was assessed using the EPDS during postpartum follow-up, conducted 6–12 weeks after delivery. The EPDS [24,25] is a validated screening instrument consisting of 10 items, each with four response options on an interval scale. Evaluation was performed by calculating the total score (minimum of 0 to a maximum of 30 points), with a cutoff of ≥10 indicating the need for further assessment of possible depression, as positive screening does not yet confirm a diagnosis of depression. The cutoff of ≥10 was applied in accordance with the threshold reported in the original validation study [26] and commonly used in subsequent clinical and epidemiological research [27,28]. Based on the total score obtained, participants were categorized into two subgroups: “elevated EPDS score” (≥10 points) vs. “non-elevated EPDS score” (0–9 points).

### 2.4. Endpoints

The primary endpoint of this study was to examine the association between metabolic parameters (HbA1c levels, insulin, C-peptide, hsCRP) measured during postpartum follow-up after pregnancies complicated by GDM and the EPDS results.

The secondary endpoint was to investigate whether specific maternal characteristics (e.g., age, BMI, smoking, employment status, breastfeeding) or neonatal outcomes (e.g., congenital anomalies, birth trauma, or postpartum transfer to NICU) act as confounding factors influencing the relationship between metabolic parameters and the outcome of the questionnaire.

### 2.5. Statistical Analysis

Statistical analysis was performed with IBM SPSS Statistics Version 30.0 for Mac OS X and R Version 4.6.1. A Chi2 test was used to compare categorical data. Since most of the data were not normally distributed, the median and interquartile range were used for data presentation and description. To compare subgroups, nonparametric tests such as the Mann–Whitney U test and the Kruskal–Wallis test were performed to compare continuous data. A *p* value < 0.05 was considered to indicate statistical significance (2-tailed).

Receiver operating characteristic (ROC) curve analysis was performed for continuous variables that differed significantly between participants with an Edinburgh Postnatal Depression Scale (EPDS) score of 0–9 and those with a score of ≥10. The discriminatory performance of each variable was quantified by the area under the ROC curve (AUC) with 95% confidence intervals. The optimal cutoff value was determined using the maximum Youden index (sensitivity + specificity − 1) [29,30,31]. Sensitivity, specificity, positive predictive value (PPV), and negative predictive value (NPV) were calculated for the selected cutoff, presented with 95% confidence intervals according to Wilson [31,32,33].

Univariate and multivariate Firth logistic regression analyses were performed to control for potential confounding factors and explore associations. In the logistic regression analysis, HbA1c was entered as a continuous variable expressed as percentage points (%). Accordingly, the reported odds ratio represents the change in odds associated with a 1.0% increase in HbA1c. No formal adjustment for multiple comparisons was performed, as the analyses were considered exploratory and hypothesis-generating.

## 3. Results

### 3.1. Patient Characteristics and Postpartum Metabolic Outcomes

The follow-up examination was conducted at a median of 10.0 weeks postpartum (IQR 8.8–11.0 weeks). The maternal, pregnancy- and delivery-related characteristics of the study population are summarized in Table 1 and presented for the two subgroups based on EPDS scores: 86.3% with scores between 0 and 9 and 13.7% with elevated EPDS scores (≥10). Analysis of the two EPDS subgroups revealed no significant differences in maternal characteristics such as age, BMI, marital status, or level of education.

In the subgroup with elevated EPDS scores, smoking was significantly more prevalent (50.0% vs. 19.5%; *p* = 0.020). The proportion of employed women was significantly higher in the subgroup with non-elevated EPDS scores (88.5% vs. 64.3%; *p* = 0.033). No significant differences were observed between the two subgroups regarding the type of GDM treatment (diet-controlled vs. insulin-dependent) or the specific insulin regimen used in insulin-treated cases. Mode of delivery and neonatal outcomes did not differ significantly between the two subgroups.

Table 2 summarizes the postpartum parameters measured during follow-up examination.

There were no statistically significant differences between the EPDS subgroups regarding the results of the postpartum 75 g oGTT or the subsequent classification of prediabetes/diabetes status.

**Figure 2:**
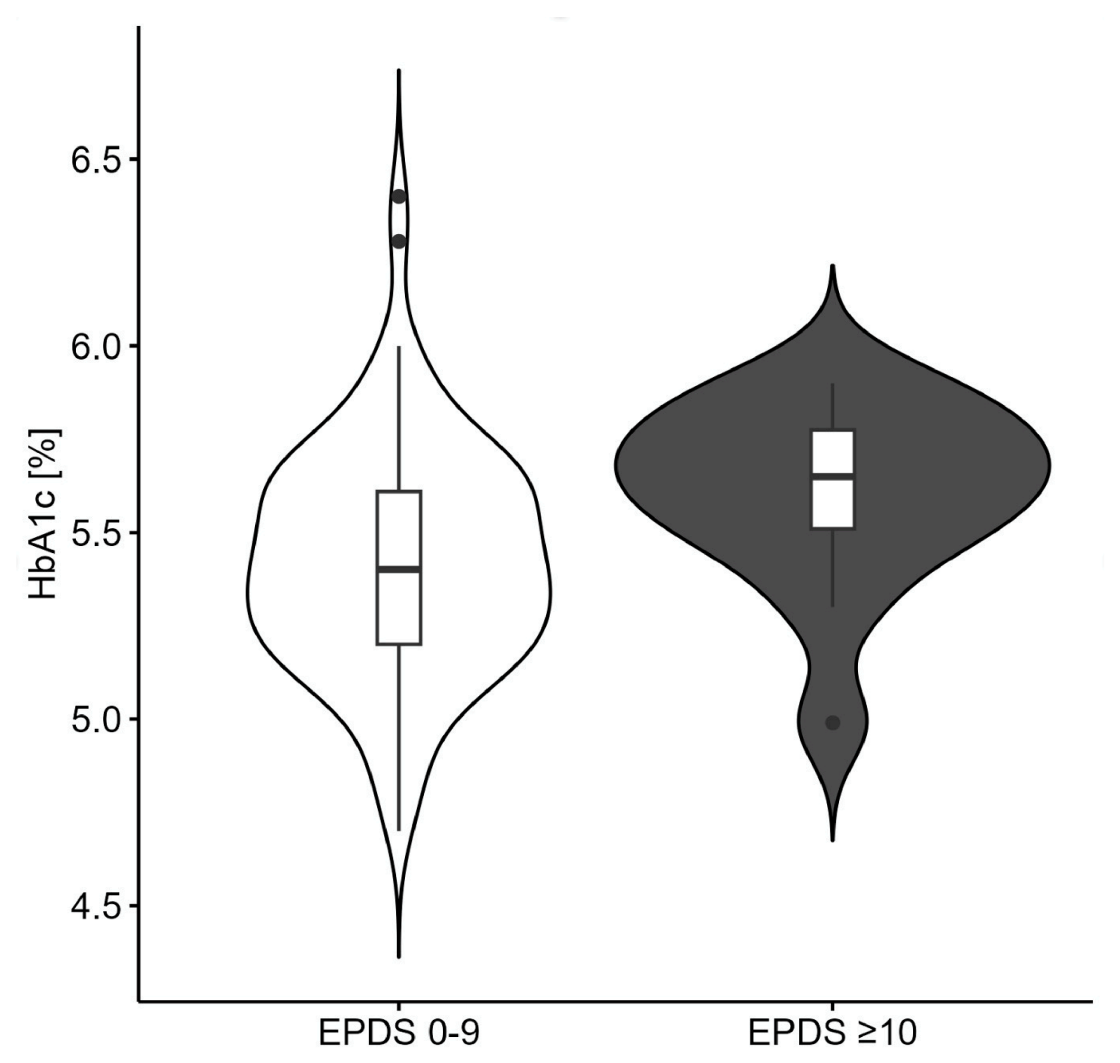
Violin plots with embedded boxplots comparing postpartum HbA1c levels between women with EPDS scores of 0–9 (*n* = 88) and those with EPDS scores ≥ 10 (*n* = 14).

HbA1c levels measured at postpartum follow-up (Figure 2) were significantly higher in the group with elevated EPDS scores [5.7% (IQR 5.5–5.8) vs. 5.4% (IQR 5.2–5.6), or 38.3 mmol/mol vs. 35.5 mmol/mol; *p* = 0.013].

There were no significant differences between the subgroups regarding calculated HOMA index or levels of insulin, C-peptide, or hsCRP. Similarly, hemoglobin levels measured at follow-up did not differ significantly between the groups.

### 3.2. Relevance of the Association Between HbA1c and Probable Depression

**Figure 3:**
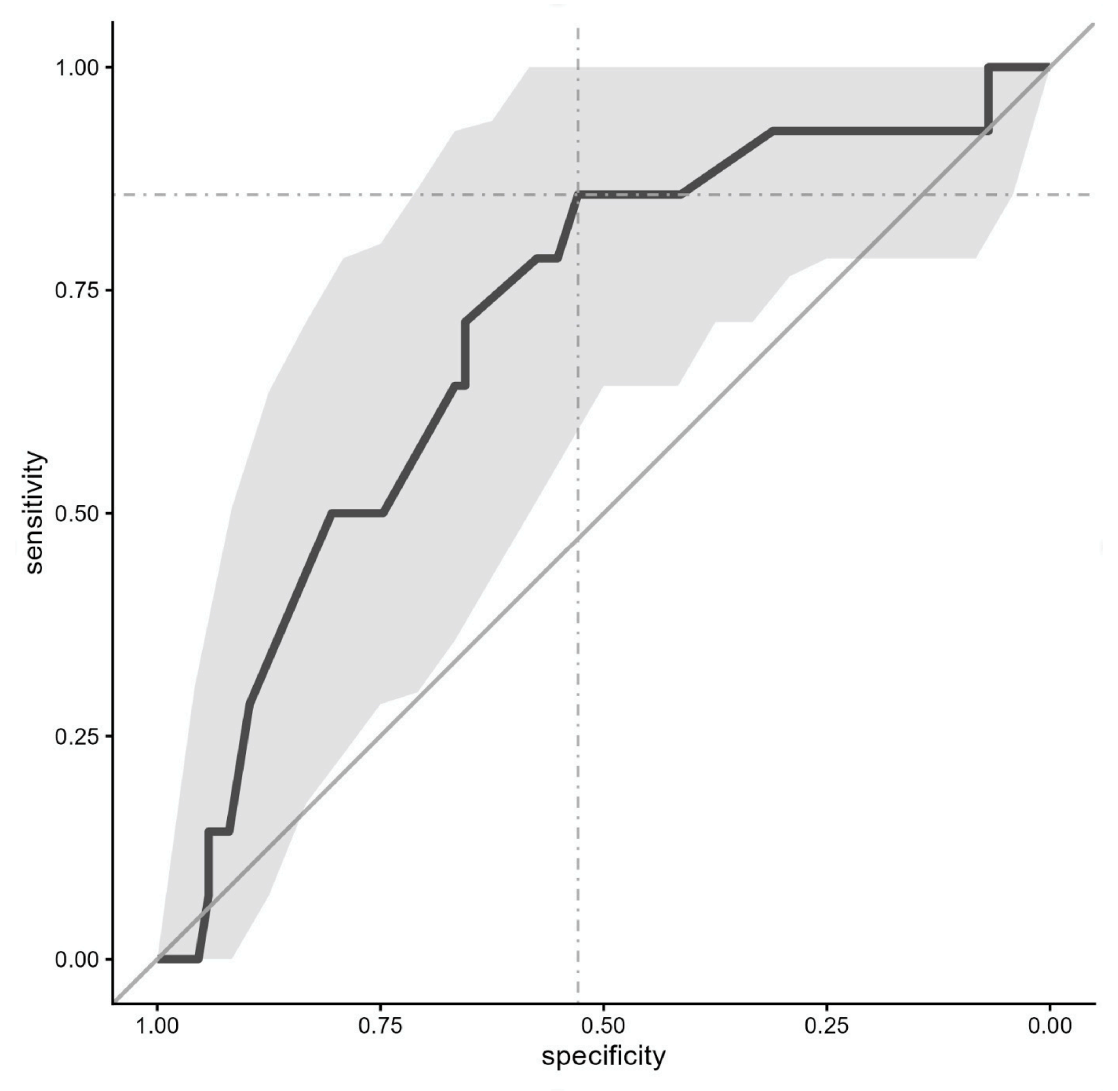
Receiver operating characteristic (ROC) curve showing the association between postpartum HbA1c (%) and elevated depressive symptoms (EPDS score ≥ 10). The shaded area represents the 95% confidence interval (CI).

The ROC analysis (Figure 3) yielded an AUC of 0.708 (95% CI: 0.567–0.849) and, using the Youden index, allowed for the identification of an HbA1c cutoff value of 5.42% (35.75 mmol/mol).

At the selected threshold, 12 cases were correctly classified as positive and 46 were correctly classified as negative. There were 2 false-negative and 41 false-positive classifications. The model achieved an accuracy of 57.4% (95% CI 47.2–67.2), with a sensitivity of 85.7% [95% CI 60.1–95.9] and a specificity of 52.9% (95% CI 42.5–63.0). The negative predictive value was 95.8% (95% CI 86.0–98.8), whereas the positive predictive value was 22.6% (95% CI 13.5–35.5). The corresponding 2 × 2 classification table is provided in Table S2 in the Supplementary Material.

### 3.3. Multivariate Analysis of Significant Factors

The results of the univariate and multivariate regression analyses for association with an elevated postpartum EPDS score (≥10) are summarized in Table 3. Univariate analyses revealed statistically significant associations for the three variables examined. A higher level of postpartum HbA1c was associated with a sixfold increased risk of an elevated postpartum EPDS score (OR = 6.666; 95% CI: 1.072–48.751; *p* = 0.042). Similarly, smoking was identified as a risk factor (OR = 4.029; 95% CI: 1.276–12.861; *p* = 0.018). In contrast, employment status appeared to be a protective factor with a substantial effect (OR = 0.234; 95% CI: 0.068–0.838; *p* = 0.026).

In the multivariable analysis, postpartum HbA1c (*p* = 0.108), which was analyzed as a continuous variable expressed as a percentage, employment status (*p* = 0.064), and smoking (*p* = 0.118) demonstrated similar directional trends as observed in the univariate analysis; however, none of these associations reached statistical significance.

## 4. Discussion

This study investigated the associations between metabolic parameters and postpartum depressive symptoms in women with pregnancies complicated by GDM, using the EPDS to identify potential subgroups with clinically relevant elevated depression scores. Women with elevated EPDS scores exhibited significantly higher HbA1c levels during the guideline-recommended screening 6–12 weeks after delivery [5.7% (IQR 5.5–5.8) vs. 5.4% (IQR 5.2–5.6), or 38.3 mmol/mol vs. 35.5 mmol/mol; *p* = 0.013]. An HbA1c cutoff value of 5.42% (35.75 mmol/mol) provided the best discrimination between women with and without elevated EPDS scores, as women with HbA1c values ≥ 5.42% were more likely to have elevated EPDS scores. This threshold demonstrated a high negative predictive value (95.8%), indicating that women with HbA1c below this cutoff were unlikely to exhibit clinically relevant depressive symptoms. Consequently, routinely measured postpartum HbA1c may serve as an accessible and routinely available marker to indicate the necessity for further psychological assessment, especially where routine depression screening has not yet been implemented. Although the negative predictive value of the exploratory HbA1c cutoff was high, this finding is strongly influenced by the relatively low prevalence of elevated EPDS scores in our study population and requires validation in larger, independent cohorts.

Importantly, HbA1c is not intended to replace routine postpartum depression screening, which should be offered to all women following GDM according to current recommendations. Rather, because postpartum HbA1c is routinely measured whereas psychological screening is not always consistently implemented, elevated HbA1c levels may represent an additional opportunity to prompt subsequent EPDS assessment in women who might otherwise remain unscreened. Nonetheless, it is important to note that the AUC showed only moderate discriminatory capacity and the positive predictive value of 22.6% limits the clinical utility of HbA1c as a standalone screening tool for postpartum depressive symptoms.

No significant associations were observed between EPDS scores and HOMA-IR or hsCRP. While previous studies in type 2 diabetes mellitus (T2DM) reported relationships between depressive symptoms, insulin resistance, and inflammation, these associations appear to be driven predominantly by somatic symptom dimensions, which are less strongly represented in the EPDS. This may explain why no comparable associations were observed in our cohort [1,6,7,34,35].

Unlike insulin levels or HOMA index, which can fluctuate depending on time of day and situational factors [36,37,38], HbA1c reflects the average blood glucose concentration over the preceding 8–12 weeks. This corresponds to the typical period in which postpartum depressive symptoms tend to emerge, as well as to the recommended 6–12-week interval for postpartum screening and metabolic follow-up. Given the observed association of higher HbA1c with elevated EPDS scores, HbA1c may therefore serve as a stable and practical complementary marker for identifying an increased risk of postpartum depression in women with a history of GDM. Although the absolute difference in HbA1c between women with and without elevated EPDS scores was modest, this finding should be interpreted in the context of our study population. As all participants received guideline-based treatment for GDM, overall glycemic control was generally good and large between-group differences were not expected. Nevertheless, even subtle differences in HbA1c may be associated with postpartum depressive symptoms and warrant further investigation in larger prospective studies in order to evaluate the clinical relevance of this observation. Notably, the median HbA1c in women with elevated EPDS scores (5.7%) in our cohort lies at the threshold of the prediabetes range [16], which may help contextualize the observed difference. No significant differences were observed when comparing hemoglobin levels at the time of postpartum follow-up, thereby ruling out persistent peripartum anemia as a confounder for lower HbA1c levels in women with non-pathological EPDS scores.

Previous studies and meta-analyses in T2DM consistently reported associations between poor glycemic control and depressive symptoms, although results remain heterogeneous due to differences in study populations and depression assessment tools. Our findings extend these observations to women with prior GDM, while highlighting the need for prospective validation [5,34,39,40,41,42,43].

Our findings extend previous research on the association between diabetes mellitus and depression during pregnancy and the postpartum period. Unlike earlier studies [3,44], the simultaneous assessment of metabolic parameters and depressive symptoms allowed a more direct evaluation of their relationship.

In the present analysis, potential confounders such as maternal age, parity, pre-pregnancy obesity, preeclampsia, mode of delivery and NICU admission leading to separation of mother and newborn were considered. These variables are frequently discussed [1,45] as possible influencing factors for the occurrence of postpartum depression.

While HbA1c, smoking, and employment status showed significant effects on elevated postpartum EPDS scores in the univariate regression analysis, none remained statistically significant in the multivariate analysis, although similar trends were observed. Therefore, our data does not provide evidence for an independent association between these variables and elevated postpartum EPDS scores. This finding underscores the complexity and multifactorial nature of the relationship between GDM and postpartum depression and indicates a trend that warrants further investigation in larger, adequately powered studies. The findings of this study should furthermore be interpreted in the context of its retrospective design.

While hemoglobin levels were available and considered in the analyses, other potential determinants of postpartum HbA1c, including peripartum or hematological factors, as well as important biological, psychological, obstetric, and psychosocial determinants of postpartum depressive symptoms were not systematically available in our dataset. Consequently, residual confounding cannot be excluded and the observed association should be interpreted as part of a multifactorial relationship rather than evidence of a predominantly biological mechanism.

A key challenge of postpartum depression screening after GDM is the management of elevated EPDS scores. In our cohort, two women reported suicidal ideation and received immediate psychological support. Although screening is often omitted in clinical practice despite guideline recommendations [10], early identification of depressive symptoms remains essential to facilitate timely support. Establishing standardized care pathways for women with elevated screening results should therefore be a priority.

Recent evidence published since 2022 has strengthened the association between GDM and postpartum depression and reinforced recommendations for routine psychological screening in this high-risk population [5]. However, the role of specific metabolic markers remains largely unexplored [3,5]. By simultaneously assessing postpartum depressive symptoms and routinely collected metabolic parameters during the recommended 6–12-week postpartum follow-up, we found that elevated HbA1c was associated with higher EPDS scores.

These findings suggest that HbA1c may serve as a pragmatic marker to identify women who should undergo additional psychological assessment if recommended postpartum depression screening has been missed. This hypothesis should be confirmed in larger prospective studies. The mechanisms underlying the observed association remain uncertain. Rather than indicating a direct effect of hyperglycemia on postpartum depressive symptoms, elevated HbA1c may reflect poorer overall metabolic health, persistent metabolic dysregulation, or lifestyle-related factors that are themselves associated with poorer mental health. Conversely, depressive symptoms may impair adherence to lifestyle recommendations, resulting in less favorable glycemic control. Because HbA1c and EPDS were assessed at the same postpartum visit, the direction of this association cannot be determined, and both explanations remain plausible.

This study has several limitations, including its retrospective, single-center design and the potential inaccuracies of electronic health records. The small cohort size, particularly the limited number of women with elevated EPDS scores, restricted adjustment for potential confounders and resulted in wide confidence intervals, limiting the precision of the effect estimates [46,47,48]. The cross-sectional design precludes conclusions regarding causality or the temporal relationship between HbA1c and postpartum depressive symptoms. In addition, the relatively low participation rate in postpartum follow-up may have introduced selection bias and limits the generalizability of our findings [49,50]. No a priori sample size calculation was performed, as the study population was defined by routine clinical follow-up, which may have reduced statistical power to detect smaller effects. Furthermore, the HbA1c cutoff was derived and evaluated within the same cohort and has not been externally validated. As this was an exploratory study, no adjustment for multiple testing was performed; therefore, the findings should be considered hypothesis-generating and confirmed in larger prospective cohorts. Finally, variation in EPDS cutoff values across populations and the predominantly Caucasian study population may limit the comparability and generalizability of our results [51].

## 5. Conclusions

This exploratory study demonstrates an association between elevated postpartum HbA1c levels and depressive symptoms 6–12 weeks after pregnancies complicated by GDM, whereas other metabolic markers were not significantly associated with EPDS scores. These findings suggest that routinely measured postpartum HbA1c may serve as a complementary marker to identify women who should undergo psychological assessment if recommended postpartum depression screening has not been performed. However, HbA1c is not a substitute for validated depression screening tools, and the proposed cutoff requires external validation before clinical application. Routine postpartum depression screening after GDM should therefore be consistently implemented to enable the early identification and timely support of women experiencing depressive symptoms. Prospective studies are needed to confirm these findings and determine whether incorporating HbA1c into postpartum follow-up can improve the identification of women at risk for postpartum depression.

## Figures and Tables

**Table 1:** Relevant baseline characteristics, pregnancy and perinatal outcome of the subgroups: EPDS 0–9 (*n* = 88) and EPDS ≥ 10 (*n* = 14). The complete presentation of the data is provided in Table S1 in the Supplementary Material.

	Parameter	EPDS 0–9 (*n* = 88; 86.3%)	EPDS ≥ 10 (*n* = 14; 13.7%)	*p*	*n* Total
Baseline Characteristics	Age (years)	34.0 (31.0–36.0)	35.0 (29.0–39.0)	0.758	102
	Pre-pregnancy BMI (kg/m^2^)	27.6 (24.6–33.6)	29.4 (23.5–34.6)	0.902	101
	Smoking	17 (19.5)	7 (50.0)	0.020 *	101
Social status	Single	5 (5.7)	1 (7.1)	0.610	102
	In a relationship	39 (44.3)	8 (57.1)	0.610	102
	Married	44 (50.0)	5 (35.7)	0.610	102
	Employed	77 (88.5)	9 (64.3)	0.033 *	101
Level of education	Higher education entrance qualification	38 (43.7)	6 (42.9)	0.180	101
	Secondary school	47 (54.0)	8 (57.1)	0.180	101
	No school-leaving certificate	2 (2.3)	-	0.180	101
	University	25 (29.1)	5 (35.7)	0.075	100
	Vocational school	9 (10.5)	2 (14.3)	0.075	100
	Vocational training	48 (55.8)	4 (28.6)	0.075	100
	No degree	4 (4.7)	3 (21.4)	0.075	100
GDM-Therapy	Diet only	45 (51.1)	8 (57.1)	0.676	102
	Insulin-treated	43 (48.9)	6 (42.9)	0.676	102
	Metformin	1 (1.1)	0 (0)	1.00	102
Maternal outcome	Spontaneous delivery	61 (69.3)	9 (64.3)	0.760	102
	Vaginal operative delivery	7 (8.0)	0 (0)	0.589	102
	C-section	20 (22.7)	5 (35.7)	0.323	102
	Induction of labor	33 (37.5)	9 (64.3)	0.059	102
	Hypertensive disorders in pregnancy	3 (3.4)	2 (14.3)	0.140	101
	HbA1c at delivery (%)	5.4 (5.2–5.6)	5.5 (5.4–5.6)	0.368	73
	HbA1c at delivery (mmol/mol)	35.5 (33.3–37.9)	35.5 (35.0–38.0)	0.657	72
Neonatal outcome	Admission to NICU	4 (4.5)	0 (0)	1.00	102

Data are *n* (%) or median (interquartile range) unless otherwise specified. *p* * Comparison of EPDS 0–9 vs. EPDS ≥ 10; *p* < 0.05 is significant and bold. Differences in *n* total reflect missing data for individual variables due to incomplete availability of routine clinical and laboratory measurements. BMI—body mass index; EPDS—Edinburgh postnatal depression scale; GDM—gestational diabetes mellitus; NICU—neonatal intensive care unit.

**Table 2:** Parameters measured at postpartum follow-up compared between the subgroups EPDS 0–9 and EPDS ≥ 10.

	Parameter	EPDS 0–9 (*n* = 88; 86.3%)	EPDS ≥ 10 (*n* = 14; 13.7%)	*p*	*n* Total
Maternal parameters	Interval between delivery and postpartum follow-up (weeks)	10.0 (9.0–11.0)	10.0 (8.0–12.0)	0.084	102
	Maternal weight (kg)	79.0 (67.0–95.0)	76.0 (69.0–98.0)	0.703	99
	Postpartum weight retention (kg)	2.0 (−1.0–5.8)	2.1 (−1.4–6.0)	0.991	98
	Birth-related complaints	10 (11.4)	2 (14.3)	0.668	102
	Currently breastfeeding	71 (81.6)	10 (71.4)	0.469	101
Metabolic parameters	Fasting glucose (mmol/L)	5.5 (5.2–5.8)	5.6 (5.1–6.3)	0.391	102
	1 h glucose (mmol/L)	9.0 (7.6–10.4)	9.3 (8.5–10.3)	0.231	100
	2 h glucose (mmol/L)	6.7 (5.5–7.9)	6.8 (5.7–8.3)	0.734	101
	HbA1c (%)	5.4 (5.2–5.6)	5.7 (5.5–5.8)	0.013 *	101
	HbA1c (mmol/mol)	35.5 (33.3–37.9)	38.3 (36.6–39.9)	0.013 *	101
	HbA1c ≥ 5.7%	17 (19.5)	7 (50.0)	0.020 *	101
	HOMA Index	2.3 (1.5–3.2)	2.4 (1.9–3.6)	0.415	101
	No Diabetes	38 (43.2)	6 (42.9)	0.982	102
	Prediabetes or DM	50 (56.8)	8 (57.1)	0.982	102
Laboratory parameters	Insulin (mU/L)	9.5 (6.3–13.7)	9.9 (7.1–14.8)	0.423	101
	C-Peptide (ng/mL)	2.4 (1.7–2.8)	2.3 (1.7–3.2)	0.694	101
	hsCRP (mg/L)	2.1 (1.2–4.2)	2.0 (1.3–8.4)	0.374	101
	Hb (mmol/L)	8.2 (7.9–8.6)	8.0 (7.8–8.3)	0.185	101

Data are *n* (%) or median (interquartile range) unless otherwise specified. *p* * Comparison of EPDS 0–9 vs. EPDS ≥ 10; *p* < 0.05 is significant and bold. Differences in *n* total reflect missing data for individual variables due to incomplete availability of routine clinical and laboratory measurements.

**Table 3:** Univariate and multivariate regression analyses for association with an elevated postpartum EPDS score following pregnancies complicated by gestational diabetes.

	Univariate			Multivariate		
Variable	Sign.	OR	95% CI	Sign.	OR	95% CI
HbA1c (%)	**0.042**	6.666	1.072–48.751	0.108	5.127	0.691–39.856
Employment status	**0.026**	0.234	0.068–0.838	0.064	0.277	0.075–1.079
Smoking	**0.018**	4.029	1.276–12.861	0.118	2.636	0.775–8.755

Data are presented as odds ratios (OR) with 95% confidence intervals (CI). *p* < 0.05 is significant and bold. HbA1c was modeled continuously, employment status and smoking were analyzed as dichotomous variables.

## Data Availability

The datasets used and/or analyzed during the current study are available from the corresponding author on reasonable request.

## Supplementary Materials

The following supporting information can be downloaded at: https://www.mdpi.com/article/10.3390/jcm15155919/s1. Table S1: Complete presentation of baseline characteristics, pregnancy, and perinatal outcome of the subgroups: EPDS 0–9 (*n* = 88) and EPDS ≥ 10 (*n* = 14); Table S2: 2 × 2 classification for the exploratory HbA1c cutoff of ≥5.42% for elevated postpartum EPDS scores.

## Abbreviations

The following abbreviations are used in this manuscript:

GDM	Gestational diabetes mellitus
OGTT	Oral glucose tolerance test
EPDS	Edinburgh Postnatal Depression Scale
HOMA	Homeostasis Model Assessment
IADPSG	International Association of Diabetes and Pregnancy Study Groups
BMI	Body mass index
ROC	Receiver operating characteristic
AUC	Area Under the Curve
NICU	Neonatal Intensive Care Unit
OR	Odds ratio
CI	Confidence interval
PPV	Positive predictive values
NPV	Negative predictive values
NGSP	National Glycohemoglobin Standardization Program
IFCC	International Federation of Clinical Chemistry
DDG	German Diabetes Association
NGT	Normal glucose tolerance
FPG	Fasting plasma glucose
