# Supplementary material for: Association Between HbA1c Levels and Postpartum Depressive Symptoms: Insights from Edinburgh Postnatal Depression Scale Screening After Gestational Diabetes Mellitus
Source: J Clin Med. 2026 Jul 29;15(15):5919. doi: 10.3390/jcm15155919 (PMC13466278; doi:10.3390/jcm15155919)
Supplement: Supplementary file 1 [file jcm-15-05919-s001.zip › Table S1.pdf]

**Table S1.** Complete presentation of baseline characteristics, pregnancy and perinatal outcome of the subgroups: EPDS 0-9 (*n* = 88) and EPDS ≥10 (*n* = 14).

| | **Parameter** | **EPDS 0-9 (*n* = 88; 86.3%)** | **EPDS ≥10 (*n* = 14; 13.7%)** | **p** | ***n* total** |
|---|---|---|---|---|---|
| Baseline Characteristics | Age (years) | 34.0 (31.0-36.0) | 35.0 (29.0-39.0) | 0.758 | 102 |
| | Pre-pregnancy BMI (kg/m2) | 27.6 (24.6-33.6) | 29.4 (23.5-34.6) | 0.902 | 101 |
| | Obesity (BMI>30 kg/m2) | 33 (37.9) | 7 (50.0) | 0.391 | 101 |
| | Smoking | 17 (19.5) | 7 (50.0) | **0.020*** | 101 |
| | Stopped smoking during pregnancy | 15 (88.2) | 3 (50.0) | 0.089 | 23 |
| Social status | Single | 5 (5.7) | 1 (7.1) | 0.610 | 102 |
| | In a Relationship | 39 (44.3) | 8 (57.1) | 0.610 | 102 |
| | Married | 44 (50.0) | 5 (35.7) | 0.610 | 102 |
| | Employed | 77 (88.5) | 9 (64.3) | **0.033*** | 101 |
| Level of education | Higher education entrance qualification | 38 (43.7) | 6 (42.9) | 0.180 | 101 |
| | Secondary school | 47 (54.0) | 8 (57.1) | 0.180 | 101 |
| | No school-leaving certificate | 2 (2.3) | - | 0.180 | 101 |
| | University | 25 (29.1) | 5 (35.7) | 0.075 | 100 |
| | Vocational school | 9 (10.5) | 2 (14.3) | 0.075 | 100 |
| | Vocational training | 48 (55.8) | 4 (28.6) | 0.075 | 100 |
| | No degree | 4 (4.7) | 3 (21.4) | 0.075 | 100 |
| GDM-Therapy | Diet only | 45 (51.1) | 8 (57.1) | 0.676 | 102 |
| | Insulin treated | 43 (48.9) | 6 (42.9) | 0.676 | 102 |
| | Basal insulin only | 40 (93.0) | 6 (100) | 1.00 | 49 |
| | MDI | 3 (7.0) | 0 (0) | 1.00 | 49 |
| | Max. insulin dosage IE | 24.0 (15.8-36.5) | 16.0 (6.0-68.5) | 0.474 | 48 |
| | Metformin | 1 (1.1) | 0 (0) | 1.00 | 102 |
| Maternal outcome | Spontaneous delivery | 61 (69.3) | 9 (64.3) | 0.760 | 102 |
| | Vaginal operative delivery | 7 (8.0) | 0 (0) | 0.589 | 102 |
| | C-section | 20 (22.7) | 5 (35.7) | 0.323 | 102 |
| | Planned C-section | 11 (55.0) | 2 (40.0) | 0.645 | 25 |
| | Emergency C-section | 9 (45.0) | 3 (60.0) | 0.645 | 25 |
| | Induction of labor | 33 (37.5) | 9 (64.3) | 0.059 | 102 |
| | Hypertensive Disorders in Pregnancy | 3 (3.4) | 2 (14.3) | 0.140 | 101 |
| | HbA1c at delivery (%) | 5.4 (5.2-5.6) | 5.5 (5.4-5.6) | 0.368 | 73 |
| | HbA1c at delivery (mmol/mol) | 35.5 (33.3-37.9) | 35.5 (35.0-38.0) | 0.657 | 72 |
| Neonatal outcome | Admission to NICU | 4 (4.5) | 0 (0) | 1.00 | 102 |
| | Congenital anomaly | 5 (5.7) | 0 (0) | 1.00 | 102 |
| | Birth trauma | 2 (2.3) | 0 (0) | 1.00 | 101 |
| | Shoulder dystocia | 3 (3.4) | 0 (0) | 1.00 | 102 |
| | Hyperbilirubinemia >180 µmol/l | 10 (15.2) | 1 (7.7) | 0.681 | 79 |
| | Hypoglycemia <2,0 mmol/l | 4 (5.0) | 1 (7.1) | 0.562 | 94 |
| | Respiratory distress syndrome | 1 (1.2) | 0 (0) | 1.00 | 96 |

Data are n (%) or median (interquartile range) unless otherwise specified. p* Comparison of EPDS 0-9 vs. EPDS ≥10; p<0.05 is significant and bold. Differences in *n total* reflect missing data for individual variables due to incomplete availability of routine clinical and laboratory measurements. BMI – body mass

index; EPDS – Edinburgh postnatal depression scale; GDM – gestational diabetes mellitus; MDI – Multiple daily injections; NICU – neonatal intensive care unit.
