# Supplementary material for: Association Between HbA1c Levels and Postpartum Depressive Symptoms: Insights from Edinburgh Postnatal Depression Scale Screening After Gestational Diabetes Mellitus
Source: J Clin Med. 2026 Jul 29;15(15):5919. doi: 10.3390/jcm15155919 (PMC13466278; doi:10.3390/jcm15155919)
Supplement: Supplementary file 1 [file jcm-15-05919-s001.zip › Table S2.pdf]

Table S2. 2 × 2 classification for the exploratory HbA1c cutoff of ≥5.42% for elevated postpartum EPDS scores.

	EPDS ≥10	EPDS <10	Total
**HbA1c ≥5.42%**	True positive (*n*=12)	False positive (*n*=41)	53
**HbA1c <5.42%**	False negative (*n*=2)	True negative (*n*=46)	48
**Total**	14	87	101

The cutoff was derived from ROC-analysis and should be considered exploratory; EPDS – Edinburgh postnatal depression scale

.
